# Exploiting topic modeling to boost metagenomic reads binning

**DOI:** 10.1186/1471-2105-16-S5-S2

**Published:** 2015-03-18

**Authors:** Ruichang Zhang, Zhanzhan Cheng, Jihong Guan, Shuigeng Zhou

**Affiliations:** 1 Shanghai Key Lab of Intelligent Information Processing, and School of Computer Science, Fudan University, 220 Handan Road, Shanghai 200433, China; 2 Department of Computer Science and Technology, Tongji University, 4800 Cao'an Highway, Shanghai 201804, China

**Keywords:** Metagenomics, Metagenomic data binning, Topic modeling

## Abstract

**Background:**

With the rapid development of high-throughput technologies, researchers can sequence the whole metagenome of a microbial community sampled directly from the environment. The assignment of these metagenomic reads into different species or taxonomical classes is a vital step for metagenomic analysis, which is referred to as *binning *of metagenomic data.

**Results:**

In this paper, we propose a new method *TM-MCluster *for binning metagenomic reads. First, we represent each metagenomic read as a set of "k-mers" with their frequencies occurring in the read. Then, we employ a probabilistic topic model -- the Latent Dirichlet Allocation (LDA) model to the reads, which generates a number of hidden "topics" such that each read can be represented by a distribution vector of the generated topics. Finally, as in the MCluster method, we apply SKWIC -- a variant of the classical K-means algorithm with automatic feature weighting mechanism to cluster these reads represented by topic distributions.

**Conclusions:**

Experiments show that the new method TM-MCluster outperforms major existing methods, including AbundanceBin, MetaCluster 3.0/5.0 and MCluster. This result indicates that the exploitation of topic modeling can effectively improve the binning performance of metagenomic reads.

## Introduction

Due to the limitations of biological experiments, traditional microbial genomic studies focus on individual bacterium genomes. However, microorganisms in an environment often have various functional effects on each other. For example, the diversity of microbes in humans has been shown to be associated with common diseases such as Inflammatory Bowel Disease (IBD) [1] and gastrointestinal disturbance [2]. Metagenomics (Environmental Genomics or Ecogenomics) is an area that studies the genetic materials recovered directly from environmental samples such as human guts, soil, dust from air conditioners, and so on. With the rapid development of next-generation sequencing (NGS) technologies, we can directly sequence the DNA reads of multiple species obtained from the mixed environmental DNA samples.

Metagenomic reads are from multiple microorganism genomes, and usually the microorganism species of most metagenomic reads are unknown. A crucial step in metagenomic analysis is to group DNA fragments from the same species together. This task is referred to as *binning *of metagenomic reads [3]. So far, a number of computational methods have been proposed by researchers to tackle this problem. These methods roughly fall into two major categories: *similarity-based methods *and *composition-based methods*.

*Similarity-based methods *first aligns metagenomic reads to known genomes, and then group reads according to the alignment result. One typical similarity-based method is MEGAN [4]. Obviously, this type of methods does not work when the microorganism genomes are unavailable. *Composition-based methods *usually adopt supervised techniques to assign reads to different groups. The features are directly extracted from the nucleotide sequences, including oligonucleotide frequencies, GC-content, codon usage etc. Up to now, SVM [5], naïve Bayes [6], KNN [7], Interpolated Markov model [8] etc. have been used to bin metagenomic reads. However, the performance of these methods still relies heavily on the availability of known genomes, which are used as training samples.

To overcome the drawbacks of the methods above, unsupervised or semi-supervised techniques were proposed to deal with metagenomic data from unknown species. Wu et al [9] proposed a method called AbundanceBin that extracts *k*-mers from sequence reads and bins reads based on the coverage of their *k*-mers, which can separate reads of very different abundance ratios. However, AbundanceBin does not work well when the datasets consist of reads of identical abundance ratios. Leung *et al*. [10] developed the MetaCluster 3.0 method that uses 4-mers to build the feature vectors. It first groups the reads into many small clusters by the *K*-median algorithm, then merges smaller clusters to larger ones so that sequences from species of low abundance ratios can be grouped into isolated clusters. MetaCluster 3.0 outperforms AbundanceBin on both evenly and unevenly distributed datasets with reads of 1000 bp. Later, Wang *et al*. introduced two improved versions of MetaCluster 3.0, which are MetaCluster 4.0 [11] and MetaCluster 5.0 [12], for the purpose of processing short reads. MetaCluster 4.0 can deal with short reads less than 500 bp by first concatenating short reads to longer ones based on sequence overlapping. However, it can not bin reads of low abundance. MetaCluster 5.0 is an extension of MetaCluster 4.0 for handling reads of low abundance. Recently, Wang *et al*. developed MetaCluster-TA [13], an assembly-assisted binning-based annotation tool for taxonomic annotation of reads. It assembles reads into long "virtual contigs", and then applies a method like MetaCluster 5.0 to clustering these contigs and reads, and finally assigns all resulting clusters to a taxonomy. The series of MetaCluster algorithms can automatically determine the number of clusters, which is extremely important for binning of metagenomic reads as most samples are from unknown species in real datasets.

We also recently proposed a novel unsupervised method called MCluster for binning metagenomic reads [14]. MCluster uses N-grams to extract sequence features and automatic feature weighting to improve the performance of the basic K-means clustering algorithm. Experimental results show that MCluster achieves obviously better overall performance than AbundanceBin and MetaCluster 3.0 on long metagenomic reads; while compared with MetaCluster 5.0, MCluster obtains a larger sensitivity, and a comparable yet more stable F-measure on short metagenomic reads.

In this paper, we try to boost the performance of MCluster by using probabilistic topic modeling to represent DNA sequences, and develop a new approach *TM-MCluster*, which is the abbreviation of ***T***opic ***M***odel based ***M***etagenomic reads ***Cluster***ing. The approach consists of three steps: 1) representing reads by vectors of *k*-mers with frequencies; (2) transforming the frequency vectors of reads to topic distribution vectors based on the topic model LDA [15], and 3) clustering the transformed reads by the SKWIC algorithm [16], as in MCluster [14]. We evaluate the new method with both simulated and real datasets, and compare it with four typical existing binning methods, including MetaCluster 3.0/5.0, AbundanceBin and MCluster. Experimental results show that *TM-MCluster *outperforms the four existing methods over most tested datasets.

Note that in machine learning and natural language processing, topic models are a type of statistical models for discovering the hidden "topics" in a collection of data. Topic models were originally proposed for text processing, later were extended and applied to image and audio as well as music processing. Recently, some researchers applied topic models for biological data processing, such as mining protein-protein relations from MEDLINE abstracts of biomedical literature [17,18], constructing mRNA module collections [19] and studying the functional groups of metagenomics samples [20]. However, there is no work that applies topic modeling to metagenomic reads binning.

## Methods

**Figure 1:**
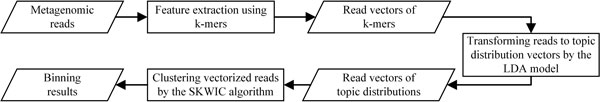
**The pipeline of the *TM-MCluster *method**.

The proposed method TM-MCluster consists of three major steps: 1) representing each read as a vector of *k*-mers with occurring frequencies; 2) transforming each read vector to a topic distribution vector based on the Latent Dirichlet Allocation (LDA) model [15]; 3) clustering the vectorized reads by the SKWIC algorithm [16], as in the MCluster method [14]. Figure 1 shows the pipeline of TM-MCluster. In what follows, we give the detail of each step.

### Representing reads by *k*-mers

Generally, the term *k*-mer refers to a sub-sequence of *k *consecutive characters in a sequence. Metagenomic data consists of many reads from different species, we use *k*-mers as the features of reads. There are at most 4^*k *^*k*-mers in a DNA sequence as there are 4 different DNA nucleotides (A,G,T,C). So a read corresponds to a vector of *k*-mers with their frequencies occurring in the read as the vector component values.

To reduce computation cost, the value of *k *should not be too large. Actually, different *k*-mers have different significance in describing DNA sequences. As shown in [21,22], *k *= 4 is the best among *k *= 2 to 7 to represent DNA sequences. So we use 4-mers to represent metagenomic reads for binning. Concretely, we slide a window of length 4 to count the frequency of each *k*-mer in a read. Here, for each 4-mer, its complementary sequence on the other chain of the DNA sequence is also considered. So the dimensionality is 256 for each read.

### Transforming reads from *k*-mer space to topic space

**Figure 2:**
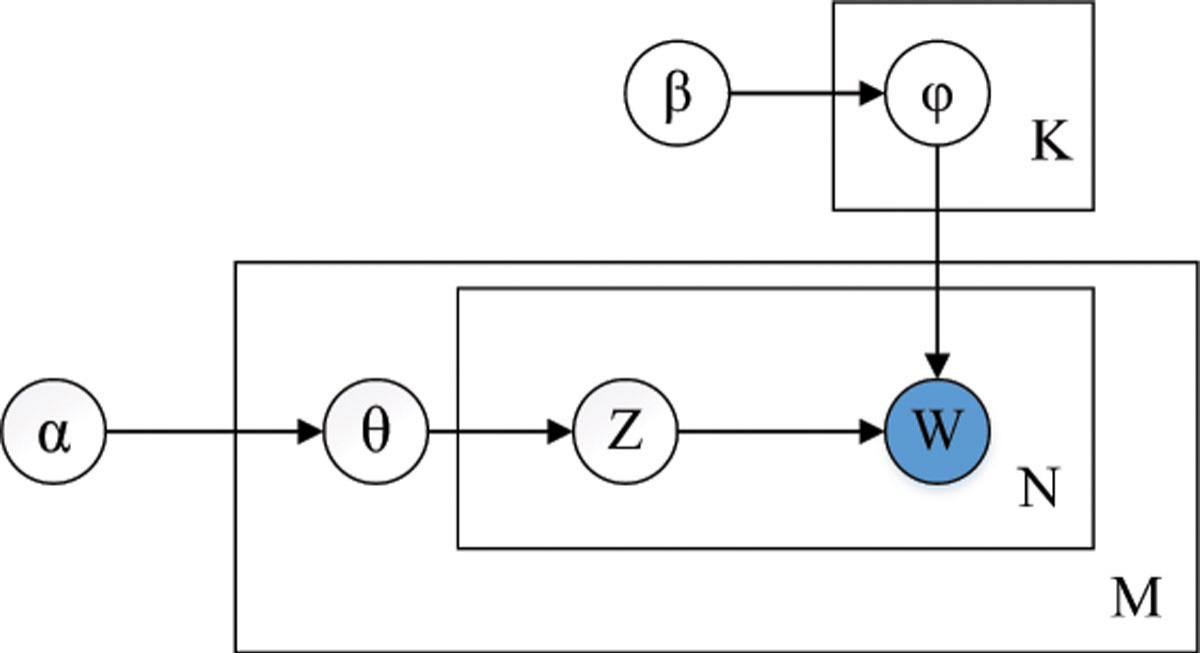
**The LDA model**.

Topic models were originally proposed to discover latent topics from a set of documents that are represented by the bag-of-word model. Latent topics indicate implicit semantic topics in documents, which have been shown to be more effective in describing the semantic relationships among documents than traditional keyword based models. In metagenomics, reads can be seen as documents and *k*-mers as keywords in documents. Reads from the same species should share more similar latent "topics" than reads from different species. So "topics" may be more effective than *k*-mers in describing metagenomic reads as far as the binning problem is considered. Here, we use the Latent Dirichlet Allocation (LDA) -- a popular topic model from machine learning area [15]. In what follows, we first introduce the LDA model, and then describe how to employ the LDA to extract latent topics from metagenomic reads. Figure 2 illustrates the LDA model. The outer plate represents documents, while the inner plate represents the repeated choice of topics and words within a document.

Some notations are as follows: *D *denotes the number of documents; *Nd *denotes the number of words in the *d*-th document; *W *denotes the number of words in the vocabulary; *T *denotes the number of topics; *α *(a *T*-dimensional vector) is the parameter of the Dirichlet prior on per-document topic distribution; *β *(a *W *- dimensional vector) is the parameter of the Dirichlet prior on per-topic word distribution; *θ_d _*(a *T*-dimensional vector) is the topic distribution for document *d *; *ϕ_j _*(a *W*-dimensional vector) is the word distribution for topic *j*;

LDA tries to generate the documents by the following process [15]:

1 For the *d*-th document, initialize *α *with random value, then choose *θ_d _*~ Dirichlet(*α*), where *d *∈ 1, 2,...,*D*;

2 For the *t*-th topic, initialize *β *with random value, then choose *ϕ_t _*~ Dirichlet(*β*);

3 For each word *w_i _*of the *d*-th document, choose *z_i _*~ Multinomial(*θ_d_*), and sample *w*_*i*_|*z_i _*~ Multinomial$\left(\phi_{z_{i}}\right)$.

We apply the LDA model to metagenomic sequences, where reads are treated as documents and *k*-mers are treated as words. Given a set of metagenomic reads, the estimation of LDA model can be estimated via the Gibbs Sampling Monte Carlo process [23]. The estimation process requires a separately sampling of latent topics for each *k*-mer in each read according to the posterior probability:

(1) $$P\left(z_{w_{i}}=j|w_{i},w_{-i},z_{-w_{i}}\right)\propto \frac{\beta +n_{-i,j}^{w_{i}}}{W\beta +n_{-i,j}^{*}}\cdot \frac{\alpha +n_{-i,j}^{d}}{T\alpha +n_{-i,*}^{d}}$$

where **w_−i _**is the current assignment *k*-mers except for *w_i_*, $z_{-w_{i}}$ is the current assignment topics of all *k*-mers except for *w_i_*, $n_{-i,j}^{w_{i}}$ is the total number of *k*-mers assigned to latent topic *j *except for the current *k*-mer *w_i_*, $n_{-i,j}^{d}$ is the total number of *k*-mers except for *w_i _*in read *d *that have been assigned to topic *j*, $n_{-i,j}^{*}$ is the total number of *k*-mers except for *w_i _*assigned to latent topic *j*, and $n_{-i,*}^{d}$ is the total number of *k*-mers except for *wi *in read *d*. In our model, we assume symmetric priors and set *α *= 0.1, *β *= 0.01. Such a parameter setting is to make topic modeling results more diverse.

**Figure 3:**
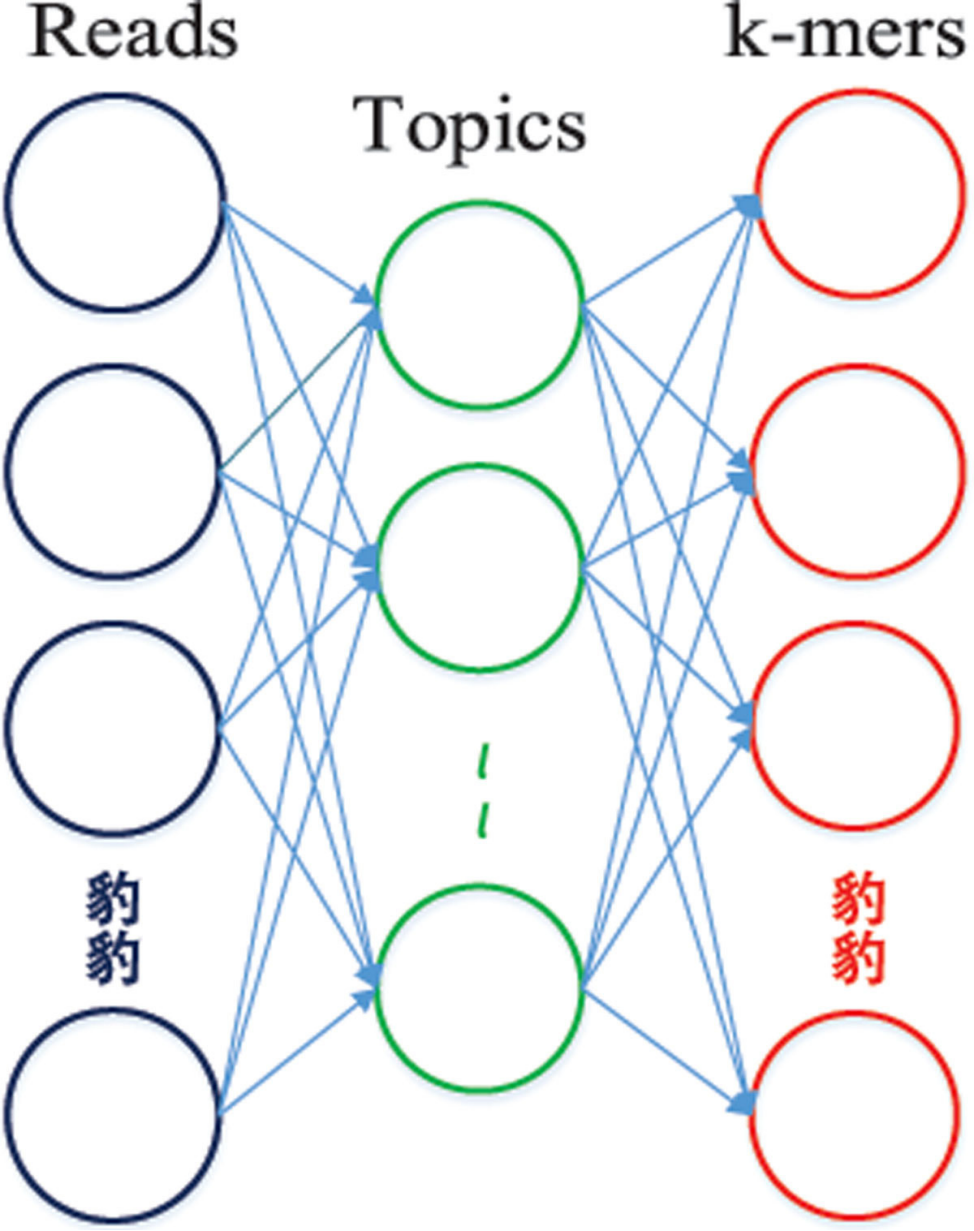
**Applying the LDA model to metagenomic reads**.

After training a LDA model, we can get the topic distribution of each read. Figure 3 illustrates the application of LDA to metagenomic reads. The left layer represents the DNA reads, the middle layer represents topics, and the right layer represents *k*-mers. We use the topic distribution of each read to represent the read. As the number of topics is usually smaller than the number of *k*-mers, this process is equivalent to dimension reduction. Here, the number of topics is a tunable parameter. In our experimental study, we set it to be 20 and 100 for simulated data and real data, respectively.

### Clustering the vectorized reads by the SKWIC algorithm

As in MCluster [14], we use the SKWIC algorithm to cluster the vectorized metagenomic reads. SKWIC is a variant of the classical *K*-means method with automatic feature weighting mechanism [16]. It tries to minimize the following objective function:

(2) $$J\left(K,V;\chi \right)=\overset{K}{\underset{i=1}{\sum }}\underset{x_{j}\in \chi_{i}}{\sum }\overset{n}{\underset{k=1}{\sum }}v_{ik}D_{wc_{ij}}^{k}+\overset{K}{\underset{i=1}{\sum }}\delta_{i}\overset{n}{\underset{k=1}{\sum }}v_{ik}^{2}$$

subject to

(3) $$v_{ik}\in \left[0,1\right]\;\forall i,k\;and\;\overset{n}{\underset{k=1}{\sum }}v_{ik}=1,\;\forall i$$

where *K *is the number of clusters, *n *is the number of dimensions, which denotes the number of topics used to represent each read here. *X_i _*is the set of reads in cluster *i*, *v_ik _*is the weight of cluster *i *in dimension *k*, $D_{wc_{ij}}^{k}$ is the distance between read *j *and the center of cluster *i *along dimension *k*.

In this objective function, we should choose a distance metric to compute the distance between reads. According to [14], Manhattan distance achieves the best performance in clustering biological sequences, compared to Euclidean and cosine distances, so here we also use Manhattan distance.

Different from the objective function of the traditional *K*-means algorithm, the objective function (2) incorporates the weight of each dimension to each cluster by *v_ik _*. *δ_i _*is used to weight the relative importance of *v_ik _*. In order to solve this optimization problem, Lagrange multiplier is adopted, and we can obtain

(4) $$v_{ik}=\frac{1}{n}+\frac{1}{2\delta_{i}}\underset{x_{j}\in \chi_{i}}{\sum }\left[\frac{\sum_{l=1}^{n}D_{wc_{ij}}^{l}}{n}-D_{wc_{ij}}^{k}\right].$$

*δ_i _*is updated iteratively as below:

(5) $$\delta_{i}^{\left(t\right)}=K_{\delta }\frac{\sum_{x_{j}\in \chi_{i}^{\left(t-1\right)}}\sum_{k=1}^{n}v_{ik}^{\left(t-1\right)}{\left(D_{wc_{ij}}^{k}\right)}^{\left(t-1\right)}}{\sum_{k=1}^{n}{\left(v_{ik}^{\left(t-1\right)}\right)}^{2}}.$$

The clustering process of SKWIC is to repeat the following steps until all cluster centroids do not change or the amount of changes is under a specified threshold:

1 Specify the number *K *of clusters (species to which the reads belong);

2 Randomly select the initial centroids of *K *clusters, and assign the weight of each dimension to each cluster equally by setting $v_{ik}=\frac{1}{n}$;

3 Iteratively update *v_ik _*by Eq. (4);

4 Assign reads to the nearest cluster;

5 Update the centroid of each cluster;

6 Update *δ_i _*by Eq. (5).

## Performance evaluation

In this section, we evaluate the performance of our method on both simulated and real datasets. We compare our method with four existing methods: MetaCluster 3.0/5.0, AbundanceBin and MCluster. MCluster is the latest unsupervised method for metagenomic data binning.

### Datasets

#### Simulated datasets

The datasets were simulated by MetaSim [24] -- a sequencing simulator for genomics and metagenomics. We generate the synthetic metagenomic datasets with sequences sampled from species of various abundance ratios.

As MetaCluster 3.0 works well only on long reads, we simulated long reads with different abundance ratios and species numbers (from 2 to 10): 16 datasets denoted by from D1 to D16; relatively-high abundance reads (50 k and 500 k reads) were also generated as AbundanceBin was designed for high abundance reads binning: 10 datasets denoted by from S1 to S10. Details of the datasets are listed in Table 1 and Table 2.

**Table 1:** Simulated datasets of low abundance (read length is 1 kbp on average).

Dataset	Reads number	Species number	Abundance ratio
D1	5k	2	1:1

D2	5k	2	1:2

D3	5k	2	1:4

D4	5k	2	1:6

D5	5k	2	1:8

D6	5k	2	1:10

D7	5k	2	1:12

D8	5k	3	1:1:1

D9	5k	3	1:3:9

D10	5k	4	1:3:3:9

D11	5k	5	1:1:1:1:1

D12	5k	5	1:1:3:3:9

D13	5k	10	1:1:1:1:1:1:1:1:1:1

D14	50k	3	1:3:9

D15	50k	4	1:3:3:9

D16	50k	5	1:1:3:3:9

**Table 2:** Simulated datasets of relatively-high abundance (read length is 1 kbp on average).

Dataset	Reads number	Species number	Abundance ratio
S1	50k	2	1:1

S2	50k	3	1:1:1

S3	50k	3	1:3:9

S4	50k	5	1:1:3:3:9

S5	50k	10	1:1:1:1:1:1:1:1:1:1

S6	500k	2	1:1

S7	500k	3	1:1:1

S8	500k	3	1:3:9

S9	500k	5	1:1:3:3:9

S10	500k	10	1:1:1:1:1:1:1:1:1:1

Nowadays, real metagenomic datasets containing millions of short reads (< 500 bp) are very common, so we simulated two datesets containing one million reads of 75 bp, belonging to 20 and 50 species respectively, which are denoted as Dataset-A and Dataset-B. Among the 20 species in Dataset-A, five species have a relative sequencing depth 1×, another five species have a relative sequencing depth 3×, the third five species have a relative sequencing depth 5×, and the remaining five species have a relative sequencing depth 10×. Among the 50 species in Dataset-B, six species have a relative sequencing depth 6×, five species have a relative sequencing depth 8×, another five species have a relative sequencing depth 10×, the remaining species have a relative sequencing depth 1×. Details of Dataset-A and Dataset-B are listed in Table 3.

**Table 3:** Simulated datasets of very high abundance (read length is 75 bp on average).

Dataset	Reads number	Species number	Abundance ratio
A	1 million	20	1 × 5:3 × 5:5 × 5:10 × 5

B	1 million	50	1 × 34:6 × 6:8 × 5:10 × 5

Considering that MetaCluster 5.0 works well only with extremely high abundance short reads, we also generated five datasets with 3000 k reads of 128 bp on average for comparing our method and MetaCluster 5.0. These datasets are denoted by C, D, E, F and G, and are presented in Table 4.

**Table 4:** Simulated datasets of extremely high abundance (read length is 128 bp on average).

Dataset	Reads number	Species number	Abundance ratio
C	3000k	2	1:1

D	3000k	3	1:1:1

E	3000k	3	1:3:9

F	3000k	5	1:1:3:3:9

G	3000k	10	1:1:1:1:1:1:1:1:1:1

#### Real dataset

**Figure 4:**
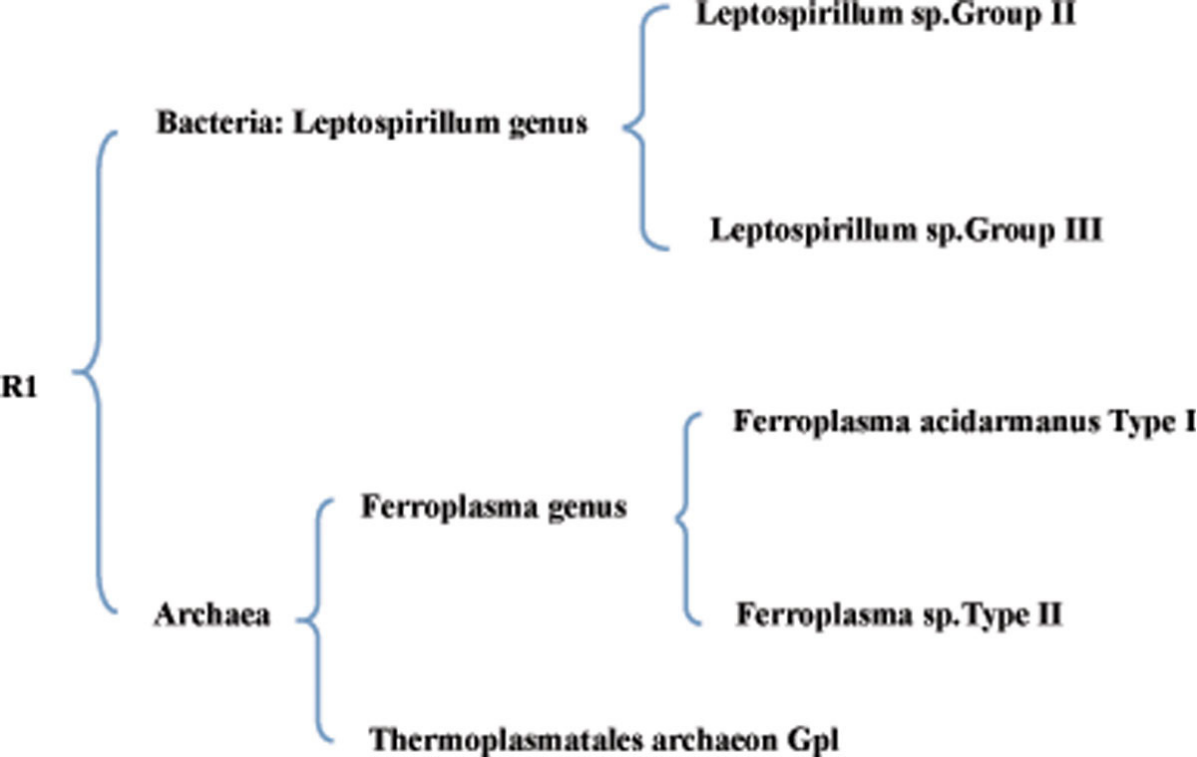
**The taxonomy of species in R1**.

We downloaded an Acid Mine Drainage metagenomics dataset from (NCBI) [25] to evaluate the performance of our method as this dataset has been well studied. The real dataset consists of 2534 contigs with an average length of 5000 bp, which were assembled by 103,462 high quality trimmed reads [26]. The dataset includes annotated sequences from 5 known species: *Leptospirillum sp.Group II*, *Leptospirillum sp.Group III*, *Ferroplasma acidarmanus Type I*, *Ferro-plasma sp.Type II *and *Thermoplasmatales archaeon Gpl*, as well as some sequences from unknown species. The five species belong to two superkingdoms and three genera, and they form the taxonomy showed in Figure 4. Since the original reads do not have species annotations, we use the 2534 annotated contigs to test the binning performance of our method. As is known, it is difficult to evaluate the performance of a binning method on sequences from unknown species, so we removed these sequences that do not belong to any known species, and eventually got 2424 contigs, which constitutes the dataset R1.

### Evaluation metrics

To evaluate the binning results, we consider three measures: Precision (*Pr*), Sensitivity (*Se*) and F1-measure (*F1*). Assume that a metagenomic dataset comes from *N *species, and finally is grouped into *M *clusters, *R_ij _*represents the number of reads in the *i*-th cluster that are from species *j*.

Precision and Sensitivity [12] are defined as follows:

(6) $$Pr=\frac{{\sum^{\text{​}}}_{i=1}^{M}{\text{max}}_{j}(R_{ij})}{{\sum^{\text{​}}}_{i=1}^{M}{\sum^{\text{​}}}_{j=1}^{N}R_{ij}},$$

(7) $$Se=\frac{\sum_{j=1}^{N}{\text{max}}_{i}\left(R_{ij}\right)}{\sum_{i=1}^{M}\sum_{j=1}^{N}R_{ij}+\text{number of unclassified reads}}.$$

Above, "unclassified reads" denotes the outliers excluded from the final result by the clustering algorithm. F1-measure [27] is defined as below:

(8) $$F1=\frac{2*Pr*Se}{Pr+Se}.$$

### Experimental results

#### The effect of topic number

**Figure 5:**
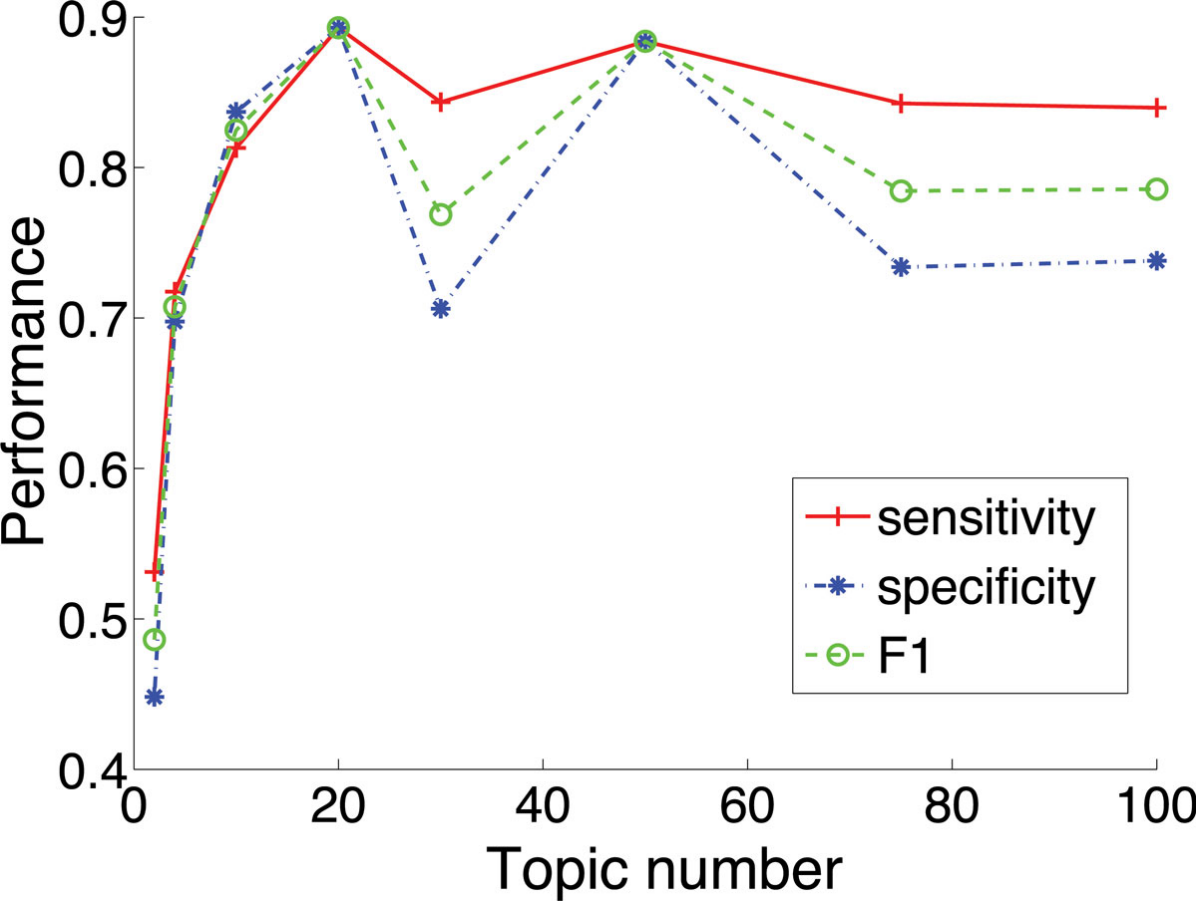
**The effect of topic number on binning performance of *TM-MCluster***.

Probabilistic topic model is an unsupervised technique. Topics in the model are latent, which means that we have to set the number of topics for a dataset as an input. Here, we check how the number of topics used in the LDA model impacts the binning performance of TM-MCluster. We use dataset D12 where the reads are from five species, and vary the topic number from 2 to 100. The results are illustrated in Figure 5. We can see that our method achieves the best overall performance when the topic number is 20. When the topic number is set to 2, the performance is the worst. It is reasonable that a too small number of topics may cause the loss of information, which thus degrades the performance. On the contrary, a too large number of topics may introduce noise, which may negatively impact the performance. From the experiments over a number of different synthetic datasets, we found that when the number of topics is set to values between 20 and 100, we can achieve satisfactory performance.

#### Results on simulated datasets

First, we compare our method with MetaCluster 3.0 and MCluster on four evenly distributed datasets D1, D8, D11 and D13 with 2, 3, 5 and 10 species, respectively. Results are shown in Table 5. As we can see in Table 5 on three of the four tested datasets, our method achieves the best *F1*; and on two of the four tested datasets, our method obtains the best *precision *or *sensitivity*.

**Table 5:** Results on simulated datasets (D1, D8, D11 and D13) with identical abundance ratio.

Dataset	MetaCluster 3.0			MCluster			TM-MCluster		
	
	*Pr*	*Se*	*F1*	*Pr*	*Se*	*F1*	*Pr*	*Se*	*F1*
D1	**.9989**	.9628	.9805	.9877	.9877	.9877	.9882	**.9882**	**.9882**

D8	.7432	.9218	.8229	.9158	.9158	.9158	**.9586**	**.9586**	**.9586**

D11	.8215	.8766	0.8481	**.9002**	**.9002**	**.9002**	.8394	.8394	.8394

D13	.4335	**.8732**	.5794	.706	.6894	.6976	**.7574**	.7732	**.7652**

Each bold value indicates the best result on a certain dataset.

We then check the performance of our method on the 12 unevenly-distributed datasets, the results are shown in Table 6. Out of the 12 tested datasets, our method achieves the best *F1*, *precision *and *sensitivity *on 10, 6 and 5 datasets, respectively; and MCluster ranks first in *F1 *and *sensitivity *on 3 datasets, while MetaCluster 3.0 outperforms the others in *precision *and *sensitivity *on 7 and 5 datasets respectively, but it does not perform best in *F*1 on any dataset. It is worth mentioning that the advantage of our method seems more outstanding on datasets with unevenly-distributed reads.

**Table 6:** Results on 12 unevenly-distributed datasets.

Dataset	MetaCluster 3.0			MCluster			TM-MCluster		
	
	*Pr*	*Se*	*F1*	*Pr*	*Se*	*F1*	*Pr*	*Se*	*F1*
D2	**.9997**	.9648	.9820	.9888	**.9888**	**.9888**	.9860	.9860	.9860

D3	**.9998**	.9596	.9793	.9950	**.9950**	**.9950**	.9948	.9948	.9948

D4	**1.0000**	.9612	.9802	.9942	.9942	.9942	.9946	**.9946**	**.9946**

D5	**1.0000**	.9608	.9800	.9950	.9950	.9950	.9954	**.9954**	**.9954**

D6	**1.0000**	.9610	.9801	.9966	**.9966**	**.9966**	.9966	**.9966**	**.9966**

D7	**1.0000**	.9618	.9805	.9980	.9980	.9980	.9988	**.9988**	**.9988**

D9	.7277	**.9628**	.8289	.8974	.8974	.8974	**.9320**	.9320	**.9320**

D10	.7345	.9096	.8127	.8852	.8852	.8852	**.9156**	**.9156**	**.9156**

D12	.7489	**.9066**	.8202	.8524	.8524	.8524	**.8930**	.8930	**.8930**

D14	.7275	**.9539**	.8255	.8863	.8860	.8863	**.9420**	.9420	**.9420**

D15	.7472	**.9202**	.8247	.8764	.8764	.8765	**.9070**	.9070	**.9070**

D16	.6792	**.9106**	.778	.8546	.8546	.8546	**.8875**	.8875	**.8875**

Each bold value indicates the best result on a certain dataset.

We go further to examine the performance of our method on datasets of relatively-high abundance. As AbundanceBin works only for high abundance datasets, here we compare our method with AbundanceBin and MCluster. The results are shown in Table 7. Out of the 10 tested datasets, our method achieves best *F1*, *sensitivity *and *precision *on 9, 6 and 8 datasets, respectively; MCluster obtains only 1 best *F1 *and 2 best *precision*; and AbundanceBin gets only 4 best *sensitivity*. This result shows that our method performs better than the other methods on high-abundance datasets.

**Table 7:** Results of on high-abundance datasets.

Dataset	AbundanceBin			MCluster			TM-MCluster		
	
	*Pr*	*Se*	*F1*	*Pr*	*Se*	*F1*	*Pr*	*Se*	*F1*
S1	.7258	.9740	.8317	.9875	.9875	.9875	**.9882**	**.9882**	**.9882**

S2	.4047	.9405	.5600	.9154	.9154	.9154	**.9519**	**.9519**	**.9519**

S3	.5866	.7528	.6594	.8873	.8873	.8873	**.9361**	**.9361**	**.9361**

S4	.4106	**.9441**	.5723	.8554	.8554	.8554	**.8921**	.8921	**.8921**

S5	.1748	**.9871**	.2970	.7361	.7241	.7301	**.7578**	.7546	**.7562**

S6	.7266	**.9999**	.8416	**.9873**	.9873	**.9873**	.9869	.9869	.9869

S7	.3991	**.9999**	.5705	.9173	.9173	.9173	**.9545**	.9545	**.9545**

S8	.8591	.8591	.8591	.8868	.8868	.8868	**.9393**	**.9393**	**.9393**

S9	.6457	.6476	.6466	.8581	.8581	.8581	**.8880**	**.8880**	**.8880**

S10	.1888	.7223	.2993	**.7253**	.7161	.7207	.7196	**.7317**	**.7256**

Each bold value indicates the best result on a certain dataset.

In reality, more and more metagenomic datasets are short reads (about 100 bp), so we also evaluate the ability of our method to deal with short metagenomic reads. As MetaCluster 3.0 does not work well on short reads, we present the results of AbundanceBin, MCluster and our method on Dataset-A and Dataset-B in Table 8. Comparing with AbundanceBin and MCluster, we can see that TM-MCluster achieves the highest F1-score and the best precision, which is roughly consistent with the results of our method on datasets of long reads.

**Table 8:** Binning performance of AbundanceBin, MCluster and TM-MCluster on short reads (75 bp average) datasets: Dataset-A and Dataset-B.

Dataset	AbundanceBin			MCluster			TM-MCluster		
	
	Pr	Se	F1	Pr	Se	F1	Pr	Se	F1
A	.2270	.9878	.3692	.2250	**1.0000**	.3674	**.3165**	.6471	**.4251**

B	.0757	.9878	.1407	.0744	**1.0000**	.1384	**.1338**	.5836	**.2177**

The bold values are the best precision, sensitivity and F1-score.

As binning large metagenomic datasets usually consumes a lot memory and time, here we present the memory and time costs of AbundanceBin, MCluster and our method on Dataset-A and Dataset-B in Table 9. We can see that AbundanceBin consumes the least memory, while MCluster runs fastest. As training LDA is time-consuming, *TM-MCluster *uses the most time and memory.

**Table 9:** Memory and time costs of AbundanceBin, MCluster and TM-MCluster on short reads (75 bp average) datasets: Dataset-A and Dataset-B.

Dataset	AbundanceBin		MCluster		TM-MCluster	
	
	Memory	Time	Memory	Time	Memory	Time
A	3.07 GB	2.15 h	3.20 GB	1.36 h	4.12 GB	3.11 h

B	3.20 GB	3.20 h	3.46 GB	2.38 h	4.10 GB	3.31 h

Finally, we compare our method TM-MCluster with MetaCluster 5.0 on datasets D, D, E, F and G in Table 4. The results are shown in Table 10 from which we can see that TM-MCluster achieves much higher sensitivity than MetaCluster 5.0 on four datasets. This is mainly because that MetaCluster 5.0 treats the reads grouped into small clusters as low abundance reads and discards them during the clustering process. But, MetaCluster 5.0 has higher precision than TM-MCluster on all the five datasets. Due to the tradeoff between precision and sensitivity, our method still obtains larger F-measure on four of the five datasets. Furthermore, MetaCluster 5.0 performs badly on the dataset D that has the largest number of species with diverse abundance ratios. In summary, our method achieves better overall performance in binning short reads than MetaCluster 5.0. d

**Table 10:** Performance comparison: TM-MCluster *vs*. MetaCluster 5.0.

Dataset	MetaCluster 5.0			TM-MCluster		
	
	*Pr*	*Se*	*F1*	*Pr*	*Se*	*F1*
C	**.9944**	.3862	.5563	.9793	**.9793**	**.9793**

D	**.9904**	.4290	.5986	.7198	.**7198**	**.7198**

E	**.9770**	**.4806**	**.6437**	.6923	.4645	.5574

F	**.9770**	.3178	.4796	.5801	**.4645**	**.5159**

D	**.8662**	.0066	.0131	.2141	**.7988**	**.3377**

#### Results on the real dataset

Here we test our method on the real dataset R1. Referring to Figure 4, we know that reads/sequences in R1 belong to two superkingdoms, three genera and five species. So we predefine the number of clusters for AbundanceBin, MCluster and our method to 2, 3 and 5, respectively. As for MetaCluster 3.0, it can automatically decide the number of final clusters, we do not predefine the cluster number. For our method, the number of latent topics is set to 100. Finally, MetaCluster 3.0 outputs two clusters. All results are presented in Table 11. Though MetaCluster 3.0 can automatically output the number of clusters, its result is not accurate, because there are five (instead of two) species in R1. For the other three methods, AbundanceBin achieves the best *sensitivity*, but its *precision *is the lowest. Our method has the best *F1*. For each input number of clusters, our method achieves the highest *F*1-score. Especially, when the number of clusters is set to 5, exactly the number of species contained in the dataset, among the four methods, our method achieves the best *precision *and *F1*, and the second highest sensitivity (only smaller than that of AbundanceBin).

**Table 11:** Results on the real dataset R1.

Methods	# **Cluster**	Pr	Se	F1
MetaCluster 3.0	2	**.7328**	.8441	.7845

AbundanceBin	2	.3952	**.9934**	.5655
	
	3	.3952	**.9934**	.5655
	
	5	.3952	**.9893**	.5648

MCluster	2	.7050	.9422	.8066
	
	3	.7054	.9179	.7978
	
	5	.6972	.6444	.6698

TM-MCluster	2	.7186	.9682	**.8250**
	
	3	**.7211**	.9645	**.8252**
	
	5	**.7182**	.9130	**.8040**

For AbundanceBin, MCluster and our method, as the input number of clusters increases from 2 to 5, the binning performance generally shows a degrading trend. The reason is like this: when the number of clusters is set to 2, 3 and 5, it is assumed to cluster the reads at superkingdom level, genus level and species level, respectively. At a higher level (e.g. superkingdom), the distance between the centers of any two clusters is generally larger than that at a lower level (e.g. species), so it is easier to group reads at a higher level than at a lower level.

## Conclusion

In this paper, we propose a new approach for binning metagenomic reads. The new approach TM-MCluster combines *k*-mer representation, topic modeling and automatic feature weighting together to boost the performance of metagenomic data binning. Experiments over both synthetic and real datasets have been conducted. The experimental results show that the new method achieves better overall performance than four existing methods, including AbundanceBin, Metacluster 3.0/5.0 and the latest MCluster method. This work indicates that the exploitation of topic modeling can effectively improve the performance of binning metagenomic sequences.

## Competing interests

The authors declare that they have no competing interests.

## Authors' contributions

Shuigeng Zhou and Jihong Guan conceived the study, and revised the manuscript. Ruichang Zhang performed all experiments and data analysis, and drafted the manuscript. Zhanzhan Cheng prepared the datasets.

## Declarations

This work was supported by China 863 Program (grant No. 2012AA020403) and National Natural Science Foundation of China (NSFC) under grants No. 61173118 and No. 61272380.

This article has been published as part of *BMC Bioinformatics *Volume 16 Supplement 5, 2015: Selected articles from the 10th International Symposium on Bioinformatics Research and Applications (ISBRA-14): Bioinformatics. The full contents of the supplement are available online at http://www.biomedcentral.com/bmcbioinformatics/supplements/16/S5.
